# Adjuvant! Online or comprehensive geriatric assessment for women over 70 years with primary breast cancer?

**DOI:** 10.1038/bjc.2012.29

**Published:** 2012-02-16

**Authors:** S Monfardini, U Basso, P Fiduccia, D Crivellari, A M Molino, C Falci

**Affiliations:** 1 Programma Oncologia Geriatrica, Istituto Palazzolo, Fondazione Don Gnocchi, Via Palazzolo 21, Milano 20149, Italy; 2 Oncologia Medica, Istituto Oncologico Veneto, via Gattamelata 64, Padova 35128, Italy; 3 Oncologia Medica C, CRO, via Pedemontana Occidentale, Aviano (PN) 33081, Italy; 4 Oncologia dO Azienda Ospedaliera Universitaria Integrata, Piazzale Stefani 1, Verona 37126, Italy

**Sir,**

We agree with the conclusions of the article on the ACTION study published in 25 October 2011 issue (Leonard *et al*, 2011). Part of the conclusions are that there is a need to explore approaches other than controlled randomised studies to evaluate the risks and benefits of chemotherapy in older patients with early breast cancer. We are also convinced, as stressed by these authors, that alternative strategies, including geriatric assessment, should be implemented.

In the absence of controlled studies in this segment of the population and on the basis of our previous monoinstitutional retrospective study pointing out at a substantial under treatment (Brunello *et al*, 2005), we embarked, starting in July 2006, in an observational study on breast cancer patients aged ⩾70 years on adjuvant therapy to shed some light on the relative usefulness of the Comprehensive Geriatric Assessment (CGA; Basso and Monfardini, 2004), and the potential role of Adjuvant! Online (ADJ) in the choice of adjuvant treatment in older breast cancer patients.

Our study was conducted in the frame of five Italians cooperating cancer centres for women with breast cancer aged ⩾70 years. All these patients were also evaluated before adjuvant therapy by the treating Physician with the CGA. Data on adjuvant therapy, clinical data and comorbidity were also entered into the ADJ program. Two hundred and seventy-two women with breast cancer, aged ⩾70 years, were observed from July 2006 to February 2009. In this series according to the CGA, interpreted as previously reported by Balducci, 47% of patients were fit, 32% vulnerable and 21% frail. Estimations of probability of non-cancer-related death and potential benefits from adjuvant treatments reported by the ADJ were blindly used by three study investigators each in three different cooperating centres (here defined as reviewers 1, 2 and 3) to express an independent therapeutic choice. Actual choices of treating Physicians, taken also on the basis of a full CGA, were then compared with that of the ADJ-based review. In 189 out of 272 patients with positive hormonal receptors, considered as higher risk because of pT ⩾2 or N+, testing the therapeutic choice of addition of Chemotherapy to endocrine therapy, the use of ADJ induced a higher percentage of choice of chemotherapy in reviewers 1 and 2 (39% and 35%, respectively) compared with the CGA-assisted choice of the treating Physician (10%).

In 18 patients with negative hormonal receptors, testing the therapeutic choice of no therapy *vs* chemotherapy, this was proposed by all the three ADJ-based reviewers in 83% of cases, with a good concordance among them, while the choice of the treating Physician of administering chemotherapy in only 61% of cases was not in accord with the choice of the three reviewers.

We cannot state that a better approach in the planning of postoperative treatment could be provided by the CGA, but these tools seems to limit the administration of adjuvant chemotherapy compared with ADJ, since according to two out of three reviewers in receptor-positive patients and three out of three reviewers in receptor-negative patients, according to the ADJ-guided choice, chemotherapy was more frequently chosen. An explanation could be that probably more obstacles to chemotherapy were encountered evaluating the patients with a multidimensional geriatric assessment.

Through the ADJ, the health status of the patient is assessed only with a limited and subjective evaluation of comorbidity, while the CGA includes an exhaustive scale such as the Cumulative Illness Rating Scale for Geriatrics. ADJ should then be used with caution and remembering that the ADJ database for patients above the age of 70 years is extremely limited. Still since in older patients the evaluation of life expectancy is crucial to make the choice on postoperative treatment, ADJ could be useful to make a rough estimation of the tumour-independent life expectancy of the patient, to know if that patient with no additional treatment will be alive without cancer or in relapse or will die for other causes.

We can then conclude that controlled studies as the ACTION, probably including only fit selected patients, should at the time being be placed side by side by observational studies. These can provide, as our study, only a limited information. But these studies, including also vulnerable and frail patients (over 50% of our patients were unfit), are more representative of the whole elderly population.
